# Supplementary material for: Spironolactone as a Potential New Treatment to Prevent Arrhythmias in Arrhythmogenic Cardiomyopathy Cell Model
Source: J Pers Med. 2023 Feb 15;13(2):335. doi: 10.3390/jpm13020335 (PMC9960914; doi:10.3390/jpm13020335)
Supplement: Supplementary file 1 [file jpm-13-00335-s001.zip › jpm-2172457-supplementary.pdf]

Supplemental Figure Legends

Supplemental Figure S1: Effect of Canrenoic acid and Spironolactone on the Action Potential paced at 1Hz. (A) Raw traces of the AP in the control condition (black)(n=27), mutated DSC2 (red)(n=32), and after incubation with CA (green)(n=43) and (B) Values for the APD at 90% repolarization. (C) Raw traces of the AP in the control condition, DSC2, and after incubation of SP (blue)(n=41) and (D) Values for the APD at 90% repolarization. Results are shown with the median and 95% confidence interval. The stars * correspond to the difference from the control. **** $p < .0001$ (Kruskal-Wallis test)

Supplemental Figure S2: Effect of Eplerenone on calcium handlings. Examples of spontaneous Ca^{2+} transient recorded in control (A) (black; n=100), DSC2 (B) (red; n=101), and DSC2+Eplerenone (C) (grey; n=98). Values of amplitude (E) and (F) the Area Under the Curve (AUC) of Ca^{2+} transients and (G) percentage of cells presenting Ca^{2+} aberrant events for the different conditions. Results are represented with the median and 95% confidence interval. The stars * correspond to the difference from the control. **** $p < .0001$ (Kruskal-Wallis test)

Supplemental Figure S1

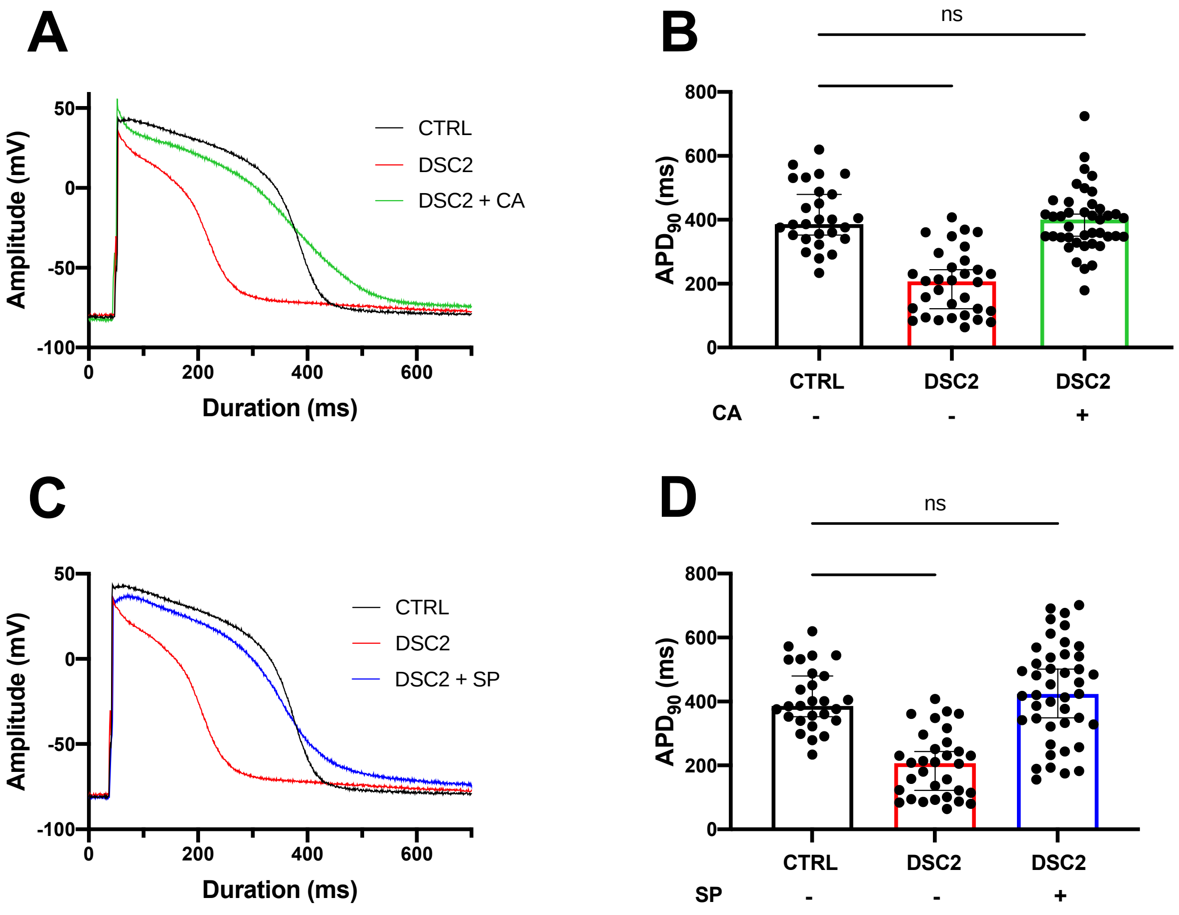

Supplemental Figure S1: Effect of Canrenoic acid and Spironolactone on the Action Potential paced at 1Hz. (A) Raw traces of the AP in the control condition (black)(n=27), mutated DSC2 (red)(n=32), and after incubation with CA (green)(n=43) and (B) Values for the APD at 90% repolarization. (C) Raw traces of the AP in the control condition, DSC2, and after incubation of SP (blue)(n=41) and (D) Values for the APD at 90% repolarization. Results are shown with the median and 95% confidence interval. The stars * correspond to the difference from the control. **** $p < .0001$ (Kruskal-Wallis test)

Supplemental Figure S2

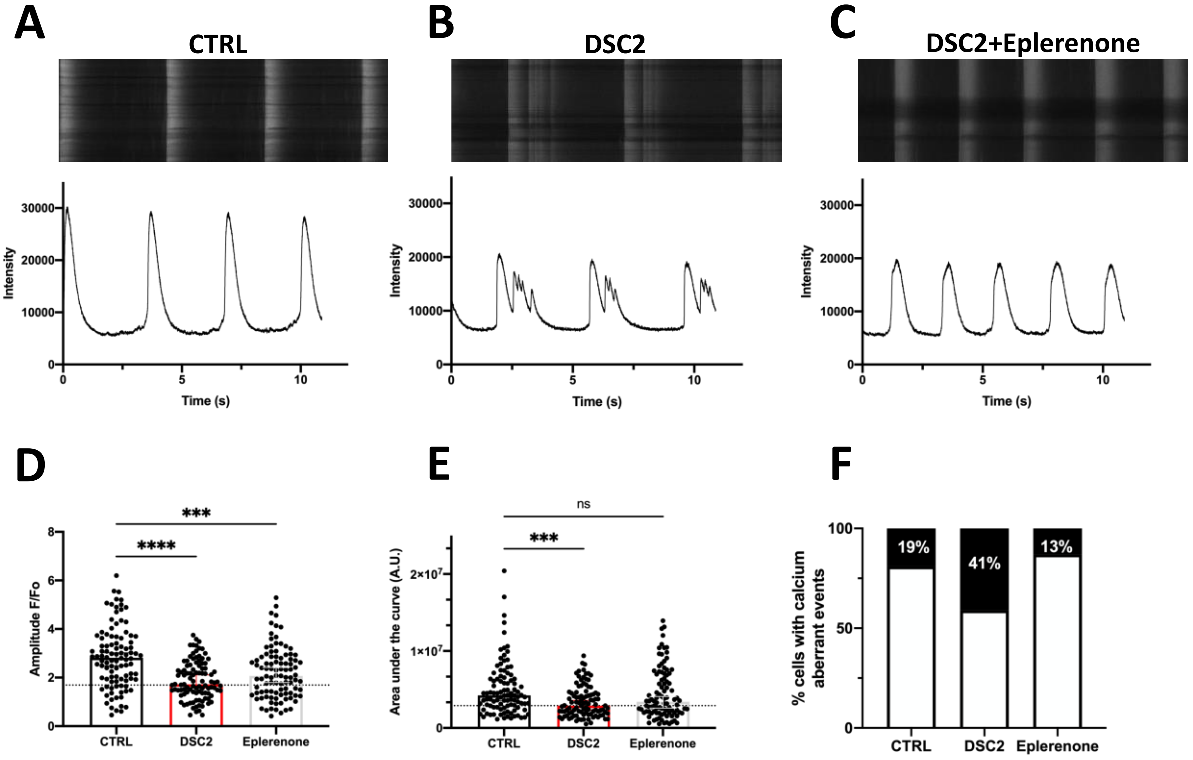

Supplemental Figure S2: Effect of Eplerenone on calcium handlings. Examples of spontaneous Ca^{2+} transient recorded in control (A) (black; n=100), DSC2 (B) (red; n=101), and DSC2+Eplerenone (C) (grey; n=98). Values of amplitude (E) and (F) the Area Under the Curve (AUC) of Ca^{2+} transients and (G) percentage of cells presenting Ca^{2+} aberrant events for the different conditions. Results are represented with the median and 95% confidence interval. The stars * correspond to the difference from the control. **** *p* < .0001 (Kruskal-Wallis test)
